# Association between herd management practices and antimicrobial resistance in *Salmonella* spp. from cull dairy cattle in Central California

**DOI:** 10.7717/peerj.6546

**Published:** 2019-03-21

**Authors:** Richard Pereira, Deniece R. Williams, Paul Rossitto, John Adaska, Emmanuel Okello, John Champagne, Terry W. Lehenbauer, Xunde Li, Jennifer Chase, Tran Nguyen, Alda F. A. Pires, Edward R. Atwill, Sharif S. Aly

**Affiliations:** 1 Department of Population Health and Reproduction, School of Veterinary Medicine, University of California, Davis, Davis, CA, USA; 2 Veterinary Medicine Teaching and Research Center, University of California, Davis, Tulare, CA, USA; 3 California Animal Health and Food Safety Laboratory System, University of California, Davis, Tulare, CA, USA; 4 Western Institute for Food Safety and Security, University of California, Davis, Davis, CA, USA

**Keywords:** *Salmonella*, Antimicrobial resistance, Dairy cattle, Cull cows

## Abstract

**Background:**

In this study cull dairy cows from six California dairy herds were sampled seasonally over the course of a year. The objectives were to determine the prevalence of antimicrobial resistant (AMR) *Salmonella* spp. shed in cull cow feces, and the factors associated with fecal shedding of AMR and multidrug resistant (MDR) *Salmonella*.

**Methods:**

Six dairy farms located in the San Joaquin Valley of California were identified and enrolled as a convenience sample. On each dairy, and once during each of the four seasons, 10 cull cows were randomly selected for fecal sampling on the day of their removal from the herd. In addition, study personnel completed a survey based on responses of the herd manager to questions related to the previous 4 month’s herd management and the specific cattle sampled. Fecal samples were submitted to the California Animal Health and Food Safety laboratory for *Salmonella* isolation. Antimicrobial resistance was evaluated using broth microdilution method and a gram-negative assay plate following Clinical Laboratory Standards Institute (CLSI) guidelines and breakpoint references. All statistical models were survey adjusted for number of animals on sampling day.

**Results:**

A total of 62 *Salmonella* were isolated from 60 of the 239 fecal samples collected. For 12% (95% confidence interval (CI) [3–20]) of fecal samples a multidrug resistant *Salmonella* was isolated. The survey-weighted results for the two most common drug classes for which isolates were resistant were tetracycline (39%; 95% CI [27–51]) and ampicillin (18%; 95% CI [9–27]). An important finding was the identification of cephalosporin as the third most common drug class for which isolates were resistant, with ceftriaxone (10%; 95% CI [2–17]) being the most common drug associated with resistance in that class. At the cow-level, reason for culling, prior treatment with antimicrobial drugs as the reason for culling was associated with higher odds of isolating an AMR *Salmonella* isolate. At the herd-level, percent of animals monthly culled on the farm as well as number of milking cows in the herd were associated with isolation of antimicrobial resistant *Salmonella* in cull cows.

**Discussion:**

*Salmonella* isolated from fecal samples from cull cows were resistant to important antimicrobials, such as ceftriaxone. The most common drug classes for which isolates were resistant were tetracyclines and beta-lactams, with ampicillin, ceftriaxone and ceftiofur being the three most common drugs within the latter. Cow and herd level factors were associated with isolating antimicrobial resistant *Salmonella* that should be further investigated for their potential role in promoting occurrence of AMR *Salmonella*. Our results also highlight the importance of monitoring dairy cattle sent to slaughter for shedding of *Salmonella* resistant to medically important antimicrobial drugs.

## Introduction

*Salmonella*, defined as nontyphoidal *Salmonella enterica*, infections are the leading cause of foodborne hospitalizations and deaths in the U.S. (Scallan et al., 2011). Food-borne *Salmonella* outbreaks traced to ground beef between 2011 and 2013 highlight the impact of this pathogen in public health (Centers for Disease Control and Prevention (CDC), 2017). Cull dairy cows comprise approximately 18% of ground beef production in the U.S. (Ott, 1996; Varma et al., 2006). California is the leading dairy state in the US, with approximately 1.73 million dairy cows, and hence the largest source of dairy beef in the nation (California Department of Food and Agriculture, 2017). Dairy cows are an important source of *Salmonella* serovars that threaten human health, including multidrug-resistant *S.* Newport and *S.* Typhimurium (Cummings et al., 2013; Hoelzer et al., 2010).

The cost of infections is higher for drug-resistant compared to susceptible *Salmonella* strains due to greater disease severity, higher hospitalization rate, and lower treatment success rate (Centers for Disease Control and Prevention (CDC), 2013). Research evaluating factors associated with multidrug resistant (MDR) *Salmonella* in cull dairy cows is needed to develop effective on-farm management practices that can reduce selection for and spread of MDR *Salmonella* strains. A previous study estimated the prevalence of *Salmonella* between 9.6% and 93.0% in cull dairy cattle at western US slaughterhouses depending on the season and day of the week that the samples were collected (Troutt et al., 2001). Recently, a similar study of dairy cows at day of culling on California dairies estimated the preharvest prevalence of *Salmonella* shedding to be 3.4% (Abu Aboud et al., 2016). However, it is not known if antimicrobial resistant (AMR) *Salmonella* strains follow similar patterns in California dairies.

The objective of the current study was to determine the prevalence of fecal shedding of AMR nontyphoidal *S. enterica* in fecal samples of cull dairy cows from six California dairy herds visited seasonally over the course of a year, and assess cow and herd level factors associated with shedding of AMR *Salmonella*.

## Material and Methods

### Farms and sampling

The study was approved by the University of California, Davis’s Institutional Animal Care and Use Committee (protocol number 18019). Six dairy farms located in the San Joaquin Valley of California were identified and enrolled in the study as a convenience sample as described previously (Abu Aboud et al., 2016) (Table 1). Cull cows were identified for fecal sampling once during each season between 2015 and 2016, specifically during summer (July 1–September 30, 2015), fall (October 1–December 31, 2015), winter (January 1–March 31, 2016) and spring (April 1–June 30, 2016). The choice of week to sample cull cows during any of the four seasons was also by convenience. From the list of cows selected by the dairy farms for culling, 10 cows were randomly selected for fecal sampling on the day of their removal from the herd using a random number generator (Excel; Microsoft Corp., Redmond, WA, USA). Random numbers were prepared specific to the total possible number of cows being presented for sampling with a specific list for each of the sampling frames consisting of multiples of 10 ranging from 11–20 to 91–100 cows. If a producer had less than 11 cows available for sale on a given sampling day, then all cows were sampled. Individual disposable polyethylene sleeves were used to manually collect fecal samples from the rectum of randomly selected cows, and the samples were transported to the Dairy Epidemiology Lab (Aly Lab) on wet ice for processing within 2–6 h of sampling.

**Table 1:** Descriptive data for six California dairy herds enrolled in a cross-sectional study of fecal shedding of antimicrobial resistant *Salmonella* spp. in cull dairy cows.

Herd	Herd percent culled per month, % (SE)	Mean milking herd size (SE)	RHA^1^, Kg (SE)	Herd breed^2^ distribution, (%)
1	4.5	3,700	11,249	H (40%), J (60%)
2	5.3	2,800	11,754	H (100%)
3	2.4	2,500	8,940	J (100%)
4	7.6	5,200	13,410	H (97%), J (3%)
5	3.0	2,800	11,203	H (100%)
6	3.1	1,500	14,900	H (100%)
All	4.3 (0.7)	3,083 (512)	11,909 (836)	

**Notes:**

1 Rolling herd average defined as the mean milk produced per milking cow in the herd during the previous year.

2 Holstein (H) and Jersey (J) breeds.

### Questionnaire

On the day of sample collection, study personnel completed a survey based on responses of the herd manager to questions related to the previous 4 month’s herd management including herd size, breed distribution, milk production, culling rate, number of times cows were culled per month, percent of cull cows sold for beef (compared to dairy purpose), percent of cull cows condemned and reason for condemnation. Herd managers were also asked questions about the percent of cull cows in the previous 4 months that received injectable medical treatments, percent of culled cows that received injectable treatments 3 weeks prior to culling, personnel allowed to administer drugs, drug residue avoidance (use of specific drugs, observing withdrawal time, testing milk and/or urine prior to culling, or other actions), tracking of drug withdrawal periods, use of a drug inventory system and extralabel drug use (familiarity and frequency). Herd level information collected was based on farm manager recollection. In addition, a backup of the herd’s Dairy Herd Improvement software file was obtained within a week of the visit to extract cull cows’ milk production and health events data. Data from all sources were housed and linked in a relational database using dairy and cow identification, and date of sampling (Access; Microsoft Corp., Redmond, WA, USA).

### Bacteriological culture

Fecal samples were submitted to the California Animal Health and Food Safety (CAHFS) lab for *Salmonella* isolation. For each sample, one g of feces was homogenized in nine ml of Tetrathionate Broth (Hardy Diagnostics, Santa Maria, CA, USA) and incubated for 18–20 h at 37 °C. The Tetrathionate broth cultures of individual samples were inoculated on Xylose lysine tergitol 4 (XLT-4) agar plates using a cotton swab and streaked with a sterile loop. The plates were incubated for 18–24 h at 37 °C and suspect *Salmonella* colonies were identified if they had a red to pink periphery with black centers. Up to three distinct and spatially isolated suspect *Salmonella* colonies on the XLT-4 plate were selected per fecal sample and each colony was streaked onto Sheep Blood Agar for further biochemical testing. If at least one isolate was confirmed as an Salmonella isolate, the sample was labeled as Salmonella positive. For biochemical testing, the selected colonies were inoculated into urea agar slants, Motility Indole Ornithine (MIO), Citrate, O-Nitrophenyl-β-D-galactopyranoside (ONPG), Lysine Iron Agar (LIA) and Triple Sugar Iron (TSI) agar slants. Colonies were designated as suspect *Salmonella* if they were urease negative, motility positive indole negative ornithine positive (MIO), citrate positive, ONPG negative, lysine positive iron positive (LIA), dextrose fermenting, and produced H_2_S (TSI). Suspect *Salmonella* colonies were confirmed using commercial polyvalent A1 and Vi antisera (DIFCO; Becton Dickinson Co., Sparks, MD, USA) following the manufacturer’s instructions.

Isolates were stored at −80 °C until completion of sampling in 2016, at which time all the *Salmonella* isolates were thawed and cultured for minimum inhibitory concentration (MIC) determination.

### Antimicrobial susceptibility testing

*Salmonella* antimicrobial resistance was evaluated using broth microdilution method using a gram-negative Sensititre plate (CMV2AGNF) (Trek Diagnostic Systems Inc., Westlake, OH, USA) according the manufacturer’s instructions. *Escherichia coli* strain ATCC25922 was used as a quality control strain. The MIC values were the lowest concentrations of antibiotics that inhibited visible growth of bacteria. Interpretations of antibiotic resistance were set by the criteria of the MIC breakpoints recommended by the Clinical and Laboratory Standards Institute (Clinical & Laboratory Standards Institute (CLSI), 2014, 2018). A *S. enterica* serovar was defined as MDR if resistance to at least one antibiotic in each of three or more drug classes was observed (Magiorakos et al., 2012). Three drugs belonging to the gram-negative assay plate (Sensititre, Trek Diagnostic Systems, Cleveland, OH, USA), namely cefoxitin, streptomycin and gentamicin, were not classified using CLSI breakpoints. As outlined in the CLSI guideline, for *Salmonella* aminoglycosides, first- and second-generation cephalosporins, and cephamycins may appear active in vitro, but are not effective clinically and should not be reported as susceptible (Clinical & Laboratory Standards Institute (CLSI), 2018). Although descriptive data for MIC results were reported, susceptibility classification for these three drugs were not part of the analysis for factors affecting antimicrobial resistance in *Salmonella* isolates.

### Statistical analysis

Descriptive analysis of the antimicrobial susceptibility classification of *Salmonella* isolates by antimicrobial drug was performed using the FREQ procedure in SAS (SAS Institute Inc., Cary, NC, USA). Descriptive analysis of *Salmonella* positive sample distribution, resistance phenotypes, and the proportion of *Salmonella* resistant to antimicrobial drugs were also performed using the FREQ procedure. To adjust for disproportionate sampling at different sampling times and between different farms, we utilized sampling weights in our statistical analysis (Pfeffermann, 1993). Weights were calculated in SAS using DATA, and were reciprocals of the probabilities of selection for the samples collected during a sampling visit; this is in relation to the total number of animals in the sampling population, which were cows to be culled that day on that farm (SAS, 2018). The survey-weighted prevalence of *Salmonella* shedding in the population of cull dairy cattle, accounting for number of animals sampled by farm, was estimated using the SURVEYMEANS function in SAS. Results for prevalence of samples were rounded to the closest integer.

#### Cow-level factors associated with Salmonella antimicrobial resistance

Weighted Chi-square test using two by two table analysis in SAS and JMP Pro 14.0 (SAS Institute Inc., Cary, NC, USA) were used to evaluate the association between cow-level factors and a fecal sample from an individual animal being positive for *Salmonella*, specifically, models were run for *Salmonella* that were classified as MDR or not, resistant to ceftriaxone or not, resistant to ciprofloxacin or not, resistant to ampicillin or not, and resistant to tetracycline or not. The latter drugs were selected because of the higher prevalence of resistance observed to these drugs or due to their classification as medically important antimicrobials. A total of 239 individual fecal samples were included in the cow-level analysis. The cow-level factors for which association with antimicrobial resistance in *Salmonella* were evaluated are present in Table S1. Chi-square analysis was weighted to account for survey sampling for each farm on sampling day and was included using the weight statement. Statistical significance was considered when a *P* < 0.05 was observed.

The association between season and a fecal sample being positive for *Salmonella* and classified as MDR or not, resistant to ceftriaxone or not, resistant to ciprofloxacin or not, resistant to ampicillin or not, and resistant to tetracycline or not was analyzed using weighted Chi-square using two by two table analysis. For each analysis, a season was compared to all other seasons combined using a binomial variable (e.g., Winter vs Spring/Summer/Fall combined). Chi-square analysis was weighted to account for survey sampling for each farm on sampling day. Statistical significance was considered when a *P* < 0.05 was observed.

#### Herd-level factors associated with Salmonella antimicrobial resistance

For evaluation of herd-level factors associated with prevalence of resistant *Salmonella*, for each sampling a herd was classified as resistant or not to the antimicrobial drug evaluated if at least one fecal sample was classified as resistant. A total of 24 herd sampling visits were included in the herd-level analysis. Herd-level survey-weights were based on the sampling fraction of cull-cows in sampled herds, as has been previously described (Wells, Dargatz & Ott, 1996; Ott, 2003). Weighted Chi-square test using two by two table analysis was used to evaluate the association between herd-level factors and a herd being classified as having as least one fecal sample with *Salmonella* MDR or not, resistant to ceftriaxone or not, resistant to ciprofloxacin or not, resistant to ampicillin or not, and resistant to tetracycline or not. Chi-square analysis was weighted to account for survey sampling for each farm on sampling day and was included using the weight statement. The herd level factors for which association with antimicrobial resistance in *Salmonella* were evaluated are present in Table S2. Statistical significance was considered when a *P* < 0.05 was observed.

Weighted Chi-square test using two by two table analysis was used to evaluate the association between season and a herd being classified as having as least one fecal sample with *Salmonella* MDR or not, resistant to ceftriaxone or not, resistant to ciprofloxacin or not, resistant to ampicillin or not, and resistant to tetracycline or not. For each analysis, a season was compared to all other seasons. Chi-square analysis was weighted to account for survey sampling for each farm on sampling day. Statistical significance was considered when a *P* < 0.05 was observed.

## Results

### Descriptive statistics

A summary of descriptive data for farm sampled is present in Table 1. Additional details of herd management and culling practices can be found in a report on *Salmonella* shedding in cull cows from the same herds (Abu Aboud et al., 2016).

### Antimicrobial susceptibility results for *Salmonella*

A total of 239 fecal samples were collected from six different farms during the four seasons. A total of 60 fecal samples were collected for each season (10 from each farm). From these samples, a total of 62 *Salmonella* isolates were isolated from the 60 fecal samples. The only exception was for herd number 5 for a sample collected from a cow in the summer, where the cow’s identification number was erroneously entered and therefore was removed from the data set due to inability to match her with information from the farms record keeping system. The samples from this cow were culture negative for *Salmonella*. The survey-weighted prevalence of *Salmonella* positive fecal samples was 31% (95% confidence interval (CI) [26–35]). The most common drug class for which *Salmonella* isolates were resistant was tetracycline, followed by the penicillin and the cephalosporin classes (Table 2). Overall 12% (95% CI [3–20]) of *Salmonella* isolates were MDR (Table 2). Survey adjusted prevalence of *Salmonella* resistant to all 14 drugs tested as well as the MIC distribution is displayed in Table 3. The three top drugs for which isolates were resistant were tetracycline (39%; 95% CI [27–51]), ampicillin (18%; 95% CI [9–27]) and ceftriaxone (10%; 95% CI [2–17]). All isolates were susceptible to azithromycin, nalidixic acid and sulfisoxazole. AMR patterns for *Salmonella* are displayed in Table 4.

**Table 2:** Survey adjusted distribution of antimicrobial resistant findings of *Salmonella* isolates by drug class and season for the 62 *Salmonella* isolates from 239 cull cow fecal samples.

Season^1^ (N^2^)	Prevalence %^3^, (SE)^4^						
	Pens^5^	Cepha^6^	Folate^7^	Phenicol^8^	Quino^9^	Tetra^10^	MDR^11^
Fall (24)	7 (7)	0 (–)	10 (7)	0 (–)	2 (1)	36 (10)	0 (–)
Winter (18)	13 (7)	19 (8)	6 (6)	13 (7)	2 (2)	37 (9)	13 (7)
Spring (13)	42 (5)	6 (5)	12 (11)	0 (–)	18 (12)	30 (12)	18 (12)
Summer (7)	30 (18)	30 (18)	0 (–)	30 (18)	0 (–)	61 (18)	30 (18)
Total (62)	18 (4)	11 (4)	7 (4)	9 (4)	5 (5)	39 (4)	12 (4)

**Notes:**

1 Study year and seasons included summer (July 1–September 30, 2015), fall (October 1–December 31, 2015), winter (January 1–March 31, 2016), and spring (April 1–June 30, 2016).

2 Number of *Salmonella* positive samples for the referenced season.

3 Prevalence of *Salmonella*.

4 Standard error of the mean.

5 Penicillins: amoxicillin/clavulanic acid or ampicillin.

6 Cephalosporin: ceftriaxone or ceftiofur.

7 Folate: sulfisoxazole or trimethoprim/sulfamethoxazole.

8 Phenicol: chloramphenicol.

9 Quinolone: nalidixic acid or ciprofloxacin.

10 Tetracycline: tetracycline.

11 Multidrug resistance: resistant to three or more antimicrobial drug classes.

**Table 3:** Distribution of minimum inhibitory concentration (MIC) and resistant for *Salmonella* isolates (*n* = 62) by individual drug.

Antimicrobial	%R (SE)**	% Distribution of MICs (μg/ml)*														
		0	0.0015	0.015	0.12	0.25	0.5	1	2	4	8	16	32	64	256	512
Tetracycline	39 (5)	67								3	0	7	22			
Ampicillin	18 (4)	82									2	2	14			
Ceftriaxone	10 (4)	81				6	3						10			
Chloramphenicol	9 (4)								3	60	27	2	8			
Amoxicillin/clavulanic acid	9 (9)	85				2	5						8			
Ceftiofur	10 (4)					3	70	5	10	3	8					
Ciprofloxacin	5 (2)	65	2	27				6								
Trimethoprim/sulfamethoxazole	7 (4)	85			3	2	2		2	6						
Azithromycin	0.0 (0)					2				77	19	2				
Nalidixic acid	0.0 (0)						2	44	34	11	6	3				
Sulfisoxazole	0.0 (0)														100	
Cefoxitin	–***							48	40	3		2	6			
Streptomycin	–***									3	69	21		6		
Gentamycin	–***	2				19	61	11	2		3	2				

**Notes:**

Highlighted areas in blue correspond to susceptible/intermediate classification, and red highlighted area corresponds to resistant classification.

* Distribution of minimum inhibitory concentration (MIC). This distribution is not survey adjusted.

** Survey adjusted prevalence (R) and standard error of the mean (SE) for *Salmonella* resistant to the referred antimicrobial drug Survey adjusted prevalence and standard error of the mean for Salmonella resistant to the referred antimicrobial drug.

*** No CLSI breakpoints available for these drugs for *Salmonella*.

**Table 4:** Distribution of antimicrobial resistance patterns and pansusceptibility among *Salmonella* isolates cultured from 239 fecal samples.

Resistance pattern	Number of isolates (*n* = 62)	% of isolates
Tet	9	14
AugAmpXnlCroChlTet	4	6
AmpTet	2	3
Sxt	2	3
Amp	1	2
AmpCip	1	2
AmpTetSxt	1	2
AugCroChlCip	1	2
Cip	1	2
CroCipTet	1	2
TetSxt	1	2
Xnl	1	2
Pansusceptible	37	60

**Note:**

Aug, amoxicillin/clavulanic acid; Amp, ampicillin; Cip, ciprofloxacin; Chl, Chloramphenicol; Cro, ceftriaxone; Sxt, Trimethoprim/sulfamethoxazole; Tet, tetracycline; Xnl, ceftiofur.

### Cow-level factors associated with *Salmonella* antimicrobial resistance

Summer was significantly associated with detection of a fecal sample positive for *Salmonella* that was resistant to ceftriaxone (odds ratio (OR) 3.2; 95% CI [1.3–7.6]), or MDR (OR 2.3; 95% CI [1.03–5.2]) (Table 5). Spring was significantly associated with detection of a fecal sample positive for *Salmonella* that was resistant to ciprofloxacin (OR 7.7; 95% CI [2.0–28.5]), or ampicillin (OR 2.7; 95% CI [1.4–5.4]).

**Table 5:** Cow-level evaluation of the association of season and isolation of a *Salmonella* resistant to the referred antimicrobial from an individual fecal sample.

Season^2^	Ceftriaxone (6/239)^1^			Ciprofloxacin (4/239)^1^			Tetracycline (18/239)^1^			Ampicillin (9/239)^1^			MDR* (7/239)^1^		
	%^3^	OR^4^ [95% CI]	*P-*value^5^	%^3^	OR^4^ [95% CI]	*P*-value^5^	%^3^	OR^4^ [95% CI]	*P*-value^5^	%^3^	OR^4^ [95% CI]	*P*-value^5^	%^3^	OR^4^ [95% CI]	*P*-value^5^
Winter	4	1.7 [0.7–4.3]	0.19	1	0.4 [0.07–2.6]	0.35	12	1 [0.6–1.7]	0.92	4	0.6 [0.2–1.3]	0.2	4	1.3 [0.5–3.1]	0.49
Spring	2	0.47 [0.1–1.9]	0.28	5	7.7 [2.0–28.5]	**0.0003**	8	0.5 [0.3–1.07]	0.07	11	2.7 [1.4–5.4]	**0.0025**	5	1.4 [0.5–3.4]	0.48
Summer	6	3.2 [1.3–7.6]	**0.0052**	0	–	–	13	1 [0.6–1.8]	0.8	6	1.2 [0.6–2.5]	0.59	6	2.3 [1.03–5.2)	**0.036**
Fall	0	–	–	1	0.6 [0.1–3.0]	0.58	15	1.3 [0.8–2.2]	0.21	3	0.4 [0.1–1.04]	0.056	0	–	–

**Notes:**

*P*-values in bold indicates a significant difference was observed.

Analysis was conducted using weighted Chi-square.

1 Number of isolates resistant to the referred drugs and the total number of samples tested.

2 Season for which odds of isolating a antimicrobial resistant Salmonella was evaluated.

3 Survey adjusted percent of fecal samples culture positive for a *Salmonella* resistant to the referred drugs within the population of animals for which samples were collected during the referred season.

4 Odds ratio for culturing a *Salmonella* resistant to the referred drug during the referred season compared to any of the other season.

5 *P*-value for the odds ratio.

* *Salmonella* isolates identified as multidrug resistant.

– During the referred season either no animals with a culture positive for a *Salmonella* resistant to the referred drugs was isolated, or no animals with a culture negative fecal sample for *Salmonella* or a culture positive sample for *Salmonella* that is susceptible to the referred antimicrobial drug. Therefore effect of season could not be evaluated.

Cow-level factors tested for association with AMR *Salmonella* from a fecal sample are shown in Table 6. Using the two by two table analysis, the only cow-level factor associated with a lower OR for isolation of resistant *Salmonella* was culling a cow due to low milk production when compared to culling a cow due to any other reason; this was observed for both ciprofloxacin (OR 0.1; 95% CI [0.03–0.6]), and tetracycline (OR 0.4; 95% CI [0.2–0.6]) resistant isolates. All other cow-level factors for which a significant difference was observed was associated with an increase in the OR for isolating antibiotic resistant *Salmonella*. These include culling due to lameness when compared to culling for any other reason being associated with isolates resistance to ciprofloxacin (OR 14.9; 95% CI [4.0–54.8]), tetracycline (OR 2.4; 95% CI [1.3–4.4]) and ampicillin (OR 3.1; 95% CI [1.4–6.8]). Treatment of cows with ampicillin prior to culling was associated with isolates resistant to ciprofloxacin (OR 7.3; 95% CI [1.1–45.9]), and treatment with ceftiofur prior to culling being associated with isolates resistant to tetracycline (OR 2.0; 95% CI [1.05–3.8]).

**Table 6:** Evaluation of cow-level factors association with isolation of a fecal sample culture positive for *Salmonella* resistant to the referred antimicrobial.

Cow-level factors^2^	Ceftriaxone (6/239)^1^			Ciprofloxacin (4/239)^1^			Tetracycline (18/239)^1^			Ampicillin (9/239)^1^			MDR* (7/239)^1^		
	%^3^	OR^4^ [95% CI]	*P*-value^5^	%^3^	OR^4^ [95% CI]	*P*-value^5^	%^3^	OR^4^ [95% CI]	*P*-value^5^	%^3^	OR^4^ [95% CI]	*P*-value^5^	%^3^	OR^4^ [95% CI]	*P*-value^5^
Treated with^6^															
Ampicillin	7	3 [0.5–18.0]	0.18	14	7.3 [1.1–45.9]	**0.01**	25	2.4 [0.7–7.5]	0.11	0	–	–	9	2.5 [0.4–14.7]	0.27
Ceftiofur	6	2 [0.6–6.3]	0.22	0	–	–	20	2 [1.05–3.8]	**0.03**	6	1 [0.3–3.0]	0.99	6	1.6 [0.5–5.0]	0.4
Cull reason^7^															
Low milk	3	1.1 [0.4–2.8]	0.78	1	0.1 [0.03–0.6]	**0.004**	8	0.4 [0.2–0.6]	**>0.0001**	6	1.6 [0.7–3.4]	0.2	4	1.5 [0.6–3.6]	0.39
Repro**	5	0.6 [0.2–1.6]	0.31	0	–	–	15	1.5 [0.99–2.5]	0.052	4	0.59 [0.3–1.3]	0.17	4	1.1 [0.5–2.5]	0.82
Lameness	0	–	–	8	14.9 [4.0–54.8]	**>0.0001**	23	2.4 [1.3–4.4]	**0.002**	13	3.1 [1.4–6.8)	**0.002**	0	–	–
Mastitis	2	1.2 [0.3–4.9]	0.76	4	3.1 [0.7–13.8]	0.11	20	1.9 [0.96–3.7]	0.057	0	–	–	4	1 [0.2–3.9]	0.99

**Notes:**

*P*-values in bold indicates a significant difference was observed.

Analysis was conducted using weighted Chi-square.

1 Number of isolates resistant to the referred drugs and the total number of samples tested.

2 Cow levels factors for which measure of association with resistant *Salmonella* was conducted.

3 Survey adjusted percent of samples that culture positive for a *Salmonella* resistant to the referred drugs within the population of animals exposed to the referred cow-level factor.

4 Odds ratio for culturing a *Salmonella* resistant to the referred drug from animals exposed to the referred cow-level factor when compared to animals not exposed to the referred cow-level factor.

5 *P*-value for the odds ratio.

6 Cows that were treated at least once with the referred antimicrobial drug.

7 Reason for culling cow from herd.

* *Salmonella* isolates identified as multidrug resistant.

** Refers to animals culled due to poor reproductive outcomes.

– No animals with a culture positive for a *Salmonella* resistant to the referred drugs was exposed to the cow-level factor, and therefore exposure effect could not be evaluated.

### Herd-level factors associated with *Salmonella* antimicrobial resistance

Spring was significantly associated with the probability of a herd being classified as having at least one fecal sample positive for ciprofloxacin resistant Salmonella when compared to other seasons combined (OR 13.6; 95% CI [2.9–62.4]) (Table 7). Winter was significantly associated with the probability of a herd being classified as having at least one fecal sample culture positive for *Salmonella* resistant to ceftriaxone (OR 4.3; 95% CI [1.3–14.4]).

**Table 7:** Herd-level evaluation of the association between season and isolation of at least one *Salmonella* resistant to the referred antimicrobial drug from a farm during a sampling visit.

Season^1^	Ceftriaxone			Ciprofloxacin			Tetracycline			Ampicillin			MDR*		
	%^2^	OR^3^ [95% CI]	*P*-value^4^	%^2^	OR^3^ [95% CI]	*P*-value^4^	%^2^	OR^3^ [95% CI]	*P*-value^4^	%^2^	OR^3^ [95% CI]	*P*-value^4^	%^2^	OR^3^ [95% CI]	*P*-value^4^
Winter	12	4.3 [1.3–14.4]	**0.014**	2	0.3 [0.05–2.4]	0.23	16	2.6 [0.8–7.7]	0.07	10	1.0 [0.3–3.2]	0.88	12	2.5 [0.8–7.7]	0.10
Spring	3	0.6 [0.1–2.8]	0.50	9	13.6 [2.9–62.4]	**0.0005**	9	1.3 [0.4–4.2]	0.66	9	1.8 [0.5–5.9)	0.33	9	2.7 [0.8–9.1]	0.11
Summer	8	1.8 [0.5–6.2]	0.35	0	–	–	8	0.5 [0.2–1.8]	0.33	8	0.8 [0.2–2.5]	0.68	8	1.1 [0.3–3.8]	0.80
Fall	0	–	–	3	0.6 [0.1–3.3]	0.59	8	0.5 [0.1–1.5]	0.21	8	0.7 [0.2–2.1]	0.53	0	–	–

**Notes:**

*P*-values in bold indicates a significant difference was observed.

Analysis was conducted using weighted Chi-square.

1 Season evaluated.

2 Survey adjusted percent of farms for which at least one fecal sample cultured positive for a *Salmonella* with resistant to the referred antimicrobial during one of the four season samplings.

3 Odds ratio for culturing at least one *Salmonella* resistant at the herd to the referred drug for herd with the referred season when compared to any other season.

4 *P*-value for the odds ratio.

* *Salmonella* isolates identified as multidrug resistant.

– During the referred season either no animals with a culture positive for a *Salmonella* resistant to the referred drugs was isolated, or no animals with a culture negative fecal sample for Salmonella or a culture positive sample for salmonella that is susceptible to the referred antimicrobial drug. Therefore effect of season could not be evaluated.

Analysis of herd-level factors tested for association with isolating resistant *Salmonella* are shown in Table 8. Farms with number of milking cow greater than 3,000 was associated with higher odds of isolating at least one *Salmonella* resistant to tetracycline (OR 3.2; 95% CI [1.1–8.9]) when compared to farms with number of milking cows less than or equal to 3,000. Monthly percent of cows culls greater than 5% was associated with higher odds of isolating at least one *Salmonella* resistant to tetracycline (OR 9.5; 95% CI [3.1–29.0]) or ampicillin (OR 5.4; 95% CI [1.8–15.9]) when compared to farms reporting a monthly cull rate of less or equal to 5%. All herd-level factors for which a significant difference was observed was associated with an increase in the OR for isolation of resistant *Salmonella*. The selection of the cut-off point for creating the binomial variables number of milk cows in the herds was based on the mean number of milking cows in herds sampled, which was 3,083 cows (standard error (SE) 512%), with the cut-off point rounded to 3,000 (Table 1). The selection of the cut-off point for creating the binomial variable monthly cull rate was based on the mean monthly cull rate reported by farms in the study, which was 4% (SE 1%), and was rounded to 5% (Table 1).

**Table 8:** Evaluation of the association of herd-level management practices with isolation of a *Salmonella* resistant to the referred antimicrobial drug from a farm during a sampling visit.

Herd-level Factors^1^	Ceftriaxone			Ciprofloxacin			Tetracycline			Ampicillin			MDR*		
	%^2^	OR^3^ [95% CI]	*P*-value^4^	%^2^	OR^3^ [95% CI]	*P*-value^4^	%^2^	OR^3^ [95% CI]	*P*-value^4^	%^2^	OR^3^ [95% CI]	*P*-value^4^	%^2^	OR^3^ [95% CI]	*P*-value^4^
Number of milking cows^5^															
**≤3,000**	16	0.3 [0.1–1.2]	0.10	8	0.6 [0.1–2.6]	0.56	14	3.2 [1.1–8.9]	**0.01**	14	2.0 [0.7–5.5]	0.16	16	0.8 [0.3–2.3)	0.68
>3,000 (Ref)**	8			6			28			22			14		
Monthly Cull (%)^6^															
**≤5%**	12	1.08 [0.3–3.3]	0.88	5	2.6 [0.6–10.6]	0.17	10	9.5 [3.1–29.0]	**>0.0001**	10	5.4 [1.8–15.9]	**0.001**	12	2.0 [0.7–5.9]	0.17
>5% (Ref)**	11			9			32			26			17		

**Notes:**

*P*-values in bold indicates a significant difference was observed.

Analysis was conducted using weighted Chi-square.

1 Herd-levels factors for which measure of association with resistant *Salmonella* was conducted and observed to be significant for at least one antimicrobial.

2 Survey adjusted percent of farms for which at least one fecal sample cultured positive for a *Salmonella* with resistant to the referred antimicrobial for each of the referred herd-level factors.

3 Odds ratio for culturing at least one *Salmonella* resistant to the referred drug from a fecal sample collected during a farm sampling visit for the referred herd level-factor when compared to herds not having the herd-level factor.

4 *P*-value for the odds ratio.

5 A binomial variable for farms that milked 3,000 cows or less and cows that milk more than 3,000 cows.

6 A binomial variable for farm that reported culling 5% or fewer cows when compared to farms that culled more than 5% of animals on a monthly basis.

* *Salmonella* isolates identified as multidrug resistant.

** Reference toward which odds ratio is being calculated. In other words, an odds ratio above one indicate the reference value has higher odds for herds having this characteristic.

## Discussion

### Antimicrobial susceptibility results for *Salmonella*

Our study focused on AMR shedding of *Salmonella* sp. in cull cows, information regarding potential effect of sampling and microbiological methods on differences observed in *Salmonella* prevalence from samples collected from farms, as used in this study, has been previously discussed in a manuscript that focuses on epidemiology of *Salmonella* sp. in California cull dairy cattle (Abu Aboud et al., 2016). The most common drug class for which nontyphoidal *S. enterica* were resistant was tetracycline (Table 2). Similar findings have been observed in other studies, with prevalence of resistance to tetracycline varying from 13% to 44% (Cummings et al., 2013; Ray et al., 2007). Resistance to the penicillin drug class was the second most common, with ampicillin being the most common drug within that class for which *Salmonella* isolates were resistant (Table 3). Similarly, other studies have reported prevalence of resistance to ampicillin varying from 10% to 42% (Cummings et al., 2013; Ray et al., 2007). An important finding was the identification of the cephalosporin class as the third most common drug class for which *Salmonella* was resistant, with resistance to ceftriaxone and ceftiofur being the two most common drugs in that class (Table 3). Multiple reports for antimicrobial susceptibility of *Salmonella* isolated from dairy cattle in the U.S. have observed prevalence of resistance to ceftriaxone and quinolone drugs to be zero, with a higher prevalence of resistance observed for ceftiofur (14% to 20%) (Lundin et al., 2008; Ray et al., 2007). Third generation cephalosporins such as ceftriaxone, and fluoroquinolone drugs, such as ciprofloxacin, are the drugs of choice when treating severe nontyphoidal *Salmonella* infections in humans (Medalla et al., 2016). However, fluoroquinolones are not routinely prescribed for children due to possible fluoroquinolone-induced joint/cartilage toxicity, and third-generation cephalosporins are particularly important as a therapeutic option in this age group, making resistance to this drug class of greater public health relevance (Leibovitz, 2006). However, it should be noted that neither ceftriaxone nor ciprofloxacin is approved by the US Food and drug administration (FDA) for use in livestock, although other third generation cephalosporin drugs and fluoroquinolones are available for use in cattle. No fluoroquinolone drug is approved by FDA for use in lactating dairy cattle, and all extra-label uses of fluoroquinolone-class antimicrobials in food animals has been prohibited in the US since 1997 (Food and Drug Administration (FDA), 2017).

Two *Salmonella* isolates resistant to ciprofloxacin were also noted to be resistant to ceftriaxone. The lack of clarity of the role of antimicrobial drug use in cattle compared to other animals species on the spread of antimicrobial-resistant *Salmonella* to human populations highlights the need for further studies that can use molecular tools to accurately measure and evaluate and generate science based information to direct future efforts. An example is a recent study by Carroll et al. (2017) that used whole-genome sequencing to compare antimicrobial-resistant *S. enterica* serovars Typhimurium, Newport and Dublin isolated from dairy cattle and humans in Washington State and New York State (Carroll et al., 2017). Although an overlap of AMR genes between *S. enterica* isolates from dairy cattle and humans was observed in the Carroll et al. study, many genetic components resulting in AMR were confined to human isolates only, indicating that different factors may be playing a role in the emergence and spread of AMR *S. enterica* in humans and farm animals.

The survey-adjusted prevalence of *Salmonella* positive fecal samples is in agreement with other studies, including one study that collected samples from cull dairy cows at five non-fed beef slaughter establishments that received cull cows and bulls from cow-calf operations (non-fed beef), representing five regions of the United States, where they observed a non-weight adjusted prevalence of 23% (Troutt et al., 2001). A study by Brichta-Harhay et al. observed the mean prevalence of MDR *Salmonella* in cull cattle to be 17%, 12% and 0.3% for hides, pre-evisceration carcass samples, and post-intervention carcass samples, respectively. In our study we observed the average of the survey-adjusted prevalence of MDR *Salmonella* to be 12% (Table 2).

### Cow-level factors Associated with *Salmonella* antimicrobial resistance

Two seasons were found to be significantly associated with resistance to specific drugs (Table 5). During the summer, higher OR for isolating MDR *Salmonella* and *Salmonella* resistant to ceftriaxone was observed, while spring was associated with higher OR for isolation of *Salmonella* resistant to ciprofloxacin and ampicillin. Further studies are needed to explain these differences, and should focus on identifying specific factors within each season that would results in the differences observed. It should also be noted that only two *Salmonella* isolates were identified as resistant to ciprofloxacin, and a high level of uncertainty for this analysis can be noted by the wide 95% CI; therefore findings for this drug must be cautiously interpreted. Differences in shedding patterns of resistant *Salmonella* could potentially be related to differences in weather conditions or management practices on farms in the regions where our study samples were collected.

Drug records indicating treatment at least once with ampicillin versus no exposure to ampicillin resulted in significantly higher OR for isolating *Salmonella* resistant to ciprofloxacin (Table 6). As already mentioned, no fluoroquinolone drugs are available to treat lactating cows, including in an extra-label matter. Furthermore, selection of resistance to ciprofloxacin under these conditions is most probably due to co-selection, although use of quinolone drugs in non-lactating cows in the herd (e.g., calves) could potentially select for resistant to the quinolone drug class. A study evaluated ciprofloxacin resistance of *E. coli* (which as *Salmonella* is an Enterobacteriaceae) in poultry in Australia, a country that has never permitted the use of fluoroquinolone in food-producing animals (Ingram et al., 2013), observed that 30% of samples collected form poultry carried fluoroquinolone non-susceptible *E. coli*. In their study, they hypothesized that the unexpected high prevalence of resistance to ciprofloxacin was probably related to co-selection, either by a clonal dissemination of chromosomal quinolone resistance determinants (vertical co-selection), or by dissemination of mobile genetic elements conferring resistance to fluoroquinolone drugs, such as *aac(6′)-Ib-cr, qnrA*, *qnrB* and *qnrS*. In their study, specific circumstances that could result in co-selection were not evaluated or suggested. Nevertheless, a study in humans has identified an association between non-fluoroquinolone therapeutic treatments and significant increase in selection of Enterobacteriacea resistant to fluoroquinolone drugs, including the use of beta-lactam drugs (Vien et al., 2012).

Drug records indicating treatment at least once with ceftiofur versus no exposure to ceftiofur resulted in significantly higher OR for *Salmonella* isolates being resistant to tetracycline (Table 6). Previous studies have identified co-selection to resistance mechanism other than those related with resistance to cephalosporins. A study evaluating the effect of parenteral treatment of cattle with ceftiofur resulted in selection of not only *bla* CMY-2, which is a gene related with phenotypic resistance to cephalosporins drugs, but also *tet*(A), a gene related with phenotypic resistance to tetracycline (Kanwar et al., 2014).

Lower OR was observed for *Salmonella* resistant to ciprofloxacin and tetracycline in cows culled due low milk production when compared to cows culled due to any other reason (Table 6). Factors that could have corroborated for these finding are not clear, and further research is needed to identify additional factors that could corroborate for these findings.

Culling cows due to lameness as compared to culling cows for any other reason resulted in higher odds for *Salmonella* resistant to ciprofloxacin, tetracycline and ampicillin. The current study did not collect information on the specific condition that resulted in the cow being culled due to lameness. This information could provide better understating about factors that could affect selection of a treatment of a lame cow. For example, a cow diagnosed with foot rot, interdigital dermatitis or a sole ulcer may receive different types of therapeutic treatment that could affect selection and shedding of resistant bacteria (Divers & Peek, 2008). The findings from our study highlight the need to collect further treatment information to better characterize specific factors that could be affecting antimicrobial resistance shedding patterns of cows culled for lameness.

A limitation of this study includes that data related to culling and drug treatment was based on farm records, which could contain error while being entered by farm personnel. Additional limitations include that samples from individual animals were collected in a cross-sectional fashion, and therefore we cannot infer causation for factors found to be associated with presence of resistance *Salmonella* in cull dairy cows. Other potential challenges that could affect precision of data collected at the farm were potential variability on the definition of disease at each farm.

Further limitations of the statistical approach used include not simultaneously accounting for the association of multiple factors with isolation of AMR *Salmonella*. The reasons for selecting Chi-square instead of an analysis that would account for more factors in the same model include low number of isolates resistant to specific drug classes in animals that were exposed to an outcome of interest. Furthermore, the lack of consistent disease definitions between the different dairies is another factor limiting further investigation of risk factors for *Salmonella* shedding. Therefore, our results should be viewed as initial effort to identify cow-level factors associated with shedding of resistant *Salmonella*, for which further studies could focus.

### Herd-level factors associated with *Salmonella* antimicrobial resistance

At the herd-level, season was significantly associated with isolating AMR *Salmonella* (Table 7). For spring, the associations observed was similar to that observed at the cow-level analysis, with higher odds for resistance to ciprofloxacin observed during spring. Resistance to ceftriaxone, however was observed to be higher during the winter at the herd-level when compared to summer at the cow-level. As already discussed with results from cow-level association with season, additional studies are needed to elucidate factors resulting in the identified role of season.

Farms with greater than 3,000 lactating cows had significantly greater odds for isolation of at least one *Salmonella* resistant to tetracycline when compared to farms with 3,000 lactating cows or less (Table 8). Furthermore, monthly culling percent greater than 5% was observed to be associated with higher OR for selection of resistance at the herd level for tetracycline and ampicillin. As observed at the cow-level, culling due to different reasons resulted in higher odds for selection of resistance in some cases (e.g., culling due to lameness), and lower in others (e.g., culling due to low milk) (Table 6). Further studies that focus on specific factors that could better explain the associations between reasons for culling animals with greater odds or not of selection of AMR *Salmonella* are needed to outline specific factors that are associated with culling and could explain the results observed.

Our findings highlight how herd level management practices can be associated with increased shedding of AMR *Salmonella* in cull cows. Approaches based on decision-making criteria both at the individual and herd level have been proposed and demonstrated to be a potential alternative for improved economical outcome of culling decisions (Fetrow, Nordlund & Norman, 2006; Haine et al., 2017; Lehenbauer & Oltjen, 1998). Therefore, improving timely culling of cows with early or mild disease problems (or significant risk for disease) before progression to more serious disease which may require antimicrobial therapy or that could result in increased risk of ante or post-mortem condemnation of the carcass could be both economical and reduce AMR selection pressure.

A previous cross-sectional study conducted by Habing et al. (2012) conducted on 265 dairy herds in 17 states observed that some of the farm management practices associated with increased prevalence of *Salmonella* included using sprinklers or misters for heat abatement (OR 2.8; CI [1.6–4.9]), feeding anionic salts to cows (OR 1.9; CI [1.1–3.5]), and feeding ionophores to cows (OR 2.1; CI [1.2–3.7]) (Habing et al., 2012). In the Habing et al. study, they observed no significant associations between antimicrobial use and detection of AMR *Salmonella* on dairy farms, although other management practices related to manure management, including application on growing pasture or hay, and use of composted/dried manure for bedding in lactating cows were associated with significantly higher presence of at least one AMR *Salmonella* on a farm. Ways by which the Habing et al. study differed from ours include collecting samples from multiple classes of cows, as well as collecting samples from multiple states.

One factor to be aware of our study is that we only had six dairy farms for which samples were collected for four seasons for the herd-level analysis (total of 24 herd-level sampling points). Therefore, interpretation of results must take into account the number of dairy farms in the study, which could affect the external validity of the results to other dairy farms in California.

Misclassification, also known as information bias, is a very common source of bias that affects the validity of questionnaire answers (Althubaiti, 2016). Participants in our study were not blinded to the objective of the study and answers to the questionnaire administered and could have been affected by misclassification, due to answers that may have been perceived as more socially desirable. To reduce misclassification in our study, the questionnaire was carefully assembled and an internal validation process conducted. Furthermore, lack of standardized guidelines or benchmarks for many of the questions asked reduces concerns with answers biased toward a more desirable standard.

## Conclusion

*Salmonella* isolated from fecal samples from cull cows had resistance to important antimicrobials used in human medicine. The two most common drug classes for which isolates were resistant were tetracyclines and penicillins. The cephalosporin class was the third most common drug class for resistance, with ceftriaxone and ceftiofur being the two most common drugs within that class. Cow and herd level factors were associated with isolation of AMR *Salmonella*. The current study highlights the importance of continued monitoring of *Salmonella* on the farm in animals going to slaughter to better address potential selection pressure for resistance to drugs of critical importance to human health.

## Supplemental Information

10.7717/peerj.6546/supp-1 Supplemental Information 1 Number of dairy cows randomly selected for fecal sampling from the list of cows identified for culling at sampling day (n) and the number of dairy cows identified for culling at sampling day (N) by herd ID and season.

10.7717/peerj.6546/supp-2 Supplemental Information 2 Variables from questionnaire used to screen for factors associated with isolation of antimicrobial resistant *Salmonella* at herd-level.

## Additional Information and Declarations

### Data Availability

The following information was supplied regarding data availability: The de-identified data was shared for peer review only as the dairy owners did not consent to publishing it alongside the article.
