# Supplementary material for: Association between herd management practices and antimicrobial resistance in Salmonella spp. from cull dairy cattle in Central California
Source: PeerJ. 2019 Mar 21;7:e6546. doi: 10.7717/peerj.6546 (PMC6431540; doi:10.7717/peerj.6546)
Supplement: Supplemental Information 1 [file peerj-07-6546-s001.docx]

Variables from questionnaire used to screen for factors associated with isolation of antimicrobial resistant *Salmonella* at the cow-level.

| Question | Type of Variable |
| --- | --- |
| 1. For what specific reason is this cow being culled? |  |
| a. Low Milk (0=No; 1=Yes) | Binomial |
| b. Poor Reproduction (0=No; 1=Yes) | Binomial |
| c. Lameness (0=No; 1=Yes) | Binomial |
| d. Post-Surgery Complication (0=No; 1=Yes) | Binomial |
| e. Mastitis (0=No; 1=Yes) | Binomial |
| f. Metabolic Disease (0=No; 1=Yes) | Binomial |
| g. Other (0=No; 1=Yes) | Binomial |
| 2. What Antimicrobials has this cow received for the condition noted above? (question 1) |  |
| Treatment 1st Drug Treatment (0=No; 1=Yes) | Binomial for each drug used |
| Treatment 2 Drug Treatment (0=No; 1=Yes) | Binomial for each drug used |
| Treatment 3 Drug Treatment (0=No; 1=Yes) | Binomial for each drug used |
| 3. Are these antibiotics used separately or in a combination? (0=No; 1=Yes) | Binomial |
| 4. Has the cow received anti-inflammatories (e.g. Banamine)? (0=No; 1=Yes) | Binomial |
| If Yes |  |
| Name of drug (0=No; 1=Yes) | Binomial for each drug used |
| 5. Has the cow received intravenous drugs? (0=No; 1=Yes) | Binomial |
| 6. Did the cow receive no treatments? (0=No; 1=Yes) | Binomial |
