# Supplementary material for: Association between herd management practices and antimicrobial resistance in Salmonella spp. from cull dairy cattle in Central California
Source: PeerJ. 2019 Mar 21;7:e6546. doi: 10.7717/peerj.6546 (PMC6431540; doi:10.7717/peerj.6546)
Supplement: Supplemental Information 2 [file peerj-07-6546-s002.docx]

Variables from questionnaire used to screen for factors associated with isolation of antimicrobial resistant *Salmonella* at herd-level.

| Question | Variable Type |
| --- | --- |
| 1. What was your herd's average number of milking cows? | Continuous |
| 2. What was your herd's rolling herd milk production? (lbs) | Continuous |
| 3. What is the percent distribution of breed(s) in your herd? |  |
| % Holstein | Continuous |
| % Jersey | Continuous |
| % Crossbreed | Continuous |
| % Other | Continuous |
| 4. How many timer per month do you cull dairy cows (for any reason)? (1 to 4) | Categorical with multiple levels |
| 5. What are the main reasons for culling? |  |
| Performance (0=No; 1=Yes) | Binomial |
| Disease (0=No; 1=Yes) | Binomial |
| Other | Categorical with multiple levels |
| 6. What percent of your culled dairy cows were sold for beef? | Continuous |
| 7. Do you feed a special fattening diet to cull dairy cows prior to marketing? (0=No; 1=Yes) | Binomial |
| If yes: |  |
| is the special diet TMR? ( 0=No; 1=Yes) | Binomial |
| is the special diet Pasture? ( 0=No; 1=Yes) | Binomial |
| is the special diet Other? ( 0=No; 1=Yes) | Binomial |
| is the special diet None? (0=No; 1=Yes) | Binomial |
| 8. Do you house cull dairy cows prior to marketing in a pen separate from other dairy cows? (0=No; 1=Yes) | Binomial |
| 9. What percent of your herd's carcasses were condemned? |  |
| 0 | Categorical with multiple levels |
| < 1 |  |
| < 1-3 |  |
| < 10 |  |
| 10. What percent of your herd's cull dairy cows received an injection of any antibiotics within 2 ~ 3 weeks of being sold? |  |
| 0 | Categorical with multiple levels |
| ≤10 |  |
| ≥10 to 20 |  |
| 30 |  |
| 11. Who perform medical treatments to sick cows? |  |
| Veterinarian | Categorical with multiple levels |
| Dairy Manager |  |
| Staff |  |
| Other |  |
| 12. What actions do you take to avoid drug residue violation? |  |
| Avoid Drug (0=No; Yes) | Binomial |
| Withdrawal period (0=No; Yes) | Binomial |
| Testing milk urine (0=No; Yes) | Binomial |
| Other | Categorical with multiple levels |
| None (0=No; Yes) | Binomial |
| 13. How do you keep track of drug withdrawal periods post‑treatment? |  |
| Computer (0=No; Yes) | Binomial |
| Chalk Marks (0=No; Yes) | Binomial |
| Paper (0=No; Yes) | Binomial |
| Memory (0=No; Yes) | Binomial |
| Other | Categorical with multiple levels |
| 14. Do you keep a record of drug names and quantities on your dairy (inventory)? (0=No; Yes) | Binomial |
| 15. If possible, can you name three main antibiotics used on your dairy? |  |
| Antibiotic 1 | Categorical with multiple levels |
| Antibiotic 2 | Categorical with multiple levels |
| Antibiotic 3 | Categorical with multiple levels |
| 16. Are these antibiotics used separately or in a combination when injected to animals? |  |
| Separately (0=No; Yes) | Binomial |
| Combined (0=No; Yes) | Binomial |
| 17. Which treatment information do you keep track of? |  |
| Date treatment (0=No; Yes) | Binomial |
| Dose (0=No; Yes) | Binomial |
| Route (0=No; Yes) | Binomial |
| Drug expiration date (0=No; Yes) | Binomial |
| Other (0=No; Yes) | Binomial |
| 18. Are you familiar with extra‑label drug use? (0=No; 1=Yes) | Binomial |
| 19. How many times per month do you use extra‑label veterinary drugs ? | Categorical with multiple levels |
| Do not use (0=No; Yes) | Binomial |
| Do not know (0=No; Yes) | Binomial |
| 20. What extra-label drugs are used and why? | Categorical with multiple levels |
| 21. Who is allowed to administer drugs to adult cows? |  |
| Herd manager (0=No; Yes) | Binomial |
| Herdsman (0=No; Yes) | Binomial |
| Limited Designated (0=No; Yes) | Binomial |
| Anyone (0=No; Yes) | Binomial |
| Veterinary (0=No; Yes) | Binomial |
| Others | Categorical with multiple levels |
| 22. Are the dairy managers and the persons administering drugs knowledgeable or trained about antibiotic resistance? (0=No; Yes) | Binomial |
| 23. Are cows given any vaccines for prevention of enteric diseases *(Salmonella,* *E. coli*, etc.)? (0=No; 1=Yes) | Binomial |
